# Partial Removal of Sugar from Apple Juice by Nanofiltration and Discontinuous Diafiltration

**DOI:** 10.3390/membranes12070712

**Published:** 2022-07-15

**Authors:** Martina Gaglianò, Carmela Conidi, Giuseppina De Luca, Alfredo Cassano

**Affiliations:** 1 Department of Chemistry & Chemical Technologies, University of Calabria, Via P. Bucci, 87036 Rende, Italy; martina.gagliano@unical.it; 2 Institute on Membrane Technology, ITM-CNR, Via P. Bucci, 17/C, 87036 Rende, Italy; c.conidi@itm.cnr.it

**Keywords:** apple juice, nanofiltration, diafiltration, total phenolic content (TPC), sugar reduction

## Abstract

Partial removal of sugars in fruit juices without compromising their biofunctional properties represents a significant technological challenge. The current study was aimed at evaluating the separation of sugars from phenolic compounds in apple juice by using three different spiral-wound nanofiltration (NF) membranes with a molecular weight cut-off (MWCO) in the range of 200–500 Da. A combination of diafiltration and batch concentration processes was investigated to produce apple juice with reduced sugar content and improved health properties thanks to the preservation and concentration of phenolic compounds. For all selected membranes, permeate flux and recovery rate of glucose, fructose, and phenolic compounds, in both diafiltration and concentration processes, were evaluated. The concentration factor of target compounds as a function of the volume reduction factor (VRF) as well as the amount of adsorbed compound on the membrane surface from mass balance analysis were also evaluated. Among the investigated membranes a thin-film composite membrane with an MWCO of 200–300 Da provided the best results in terms of the preservation of phenolic compounds in the selected operating conditions. More than 70% of phenolic compounds were recovered in the retentate stream while the content of sugars was reduced by about 60%.

## 1. Introduction

Apples are among the most commonly consumed fruits in the world because of their availability throughout the year in a variety of products including fresh fruit, juice, concentrate, and puree [1]. Epidemiological studies have shown that apple consumption as fresh fruit is associated with a reduced risk of chronic pathologies such as cardiovascular disease, specific cancers, and diabetes [2]. These beneficial health effects are mainly attributed to their content of bioactive compounds such as phytochemicals, vitamin C, dietary fibers, and pectin. 

Unfortunately, processing steps for producing ready-to-drink apple juices, including juice extraction and clarification, have a negative impact on the health-promoting compounds of apples: typically, the content in polyphenol and vitamin C is reduced following processing and juicing and fibers are almost completely removed in the clarification step [3]. In addition, clear apple juice has been associated with adverse effects, mainly related to its high fructose and low fiber content [4]. Similar to sweetened beverages, apple juice contains, on average, 9.6 g of sugar in 100 g.

New World Health Organization (WHO) guidelines recommend that adults and children reduce their daily intake of free sugars to less than 10% of their total energy intake [5]. A further reduction to less than 5% of their total caloric intake—equivalent to about 25 g of sugar per day for a person with a healthy body weight—provides additional health benefits [6].

In addition, in the last years, consumer food requirements have been changed considerably, with a significant increase in the consumption of foods with beneficial health properties and a lower consumption of fruit juices enriched in sugar [7]. Therefore, the use of technological methodologies for the partial removal of sugar from apple juice, thus limiting the free sugar intake without compromising its biofunctional properties mainly attributed to phenolic acids and polyphenols [8], appears to be a strategy of great interest for both manufacturers and consumers. The idea is to get a juice with a “large portfolio” of beneficial effects. 

Although chromatographic separations continue to attract extensive interest for the target separation of sugars, these processes are difficult to apply on large scale. The alternatives may include biochemical transformations of sugars into corresponding compounds such as ethanol, oligosaccharides, or organic acids, but these would lead to a significant change in the fruit juice, which is not in keeping with the previous goal. A further possibility which, instead, allows for the separation of sugars while maintaining the unchanged metabolic composition of the juice, is represented by membrane filtration. Pressure-driven membrane operations, such as microfiltration (MF), ultrafiltration (UF), nanofiltration (NF), and reverse osmosis (RO) are currently well-established technologies for the clarification, stabilization, fractionation, and concentration steps of fruit juice production [9]. These processes offer several advantages over conventional technologies due to their mild operating conditions of temperature (therefore preserving the functional properties of food products), low operating and maintenance costs, non-use of chemical agents or solvents, and, consequently, the possibility of avoiding product contamination [10].

Their application in the industrial production of fruit and vegetable juices fits well with sustainable food processing, an integral part of the sustainable food supply chain and sustainable development based on the use of low energy and low-impact processing schemes to produce high-quality products with nutritional values close to that of fresh products [11].

In particular, the use of nanofiltration (NF) membranes has been largely investigated for the fractionation and concentration of flavonoids, anthocyanins, carotenoids, sugars, and phenolic compounds from fruit and vegetable matrices including graviola (*Annona muricata* L.) [12], jussara (*Euterpe edulis*) [13], wine lees [14], grape pomace [15], propolis [16], and roselle (*Hibiscus sabdariffa* L.) [17] extracts, as well as elderberry (*Sambucus nigra* L.) [18], pomegranate [19], bergamot [20], and apple [21] juices. Nevertheless, the metabolic composition of apple juice is quite complex and the molecular masses of valuable compounds are close to those of sugars (mono and disaccharides), which results in a membrane insufficient selectivity [22]. In order to increase the level of separation and to achieve a high purity of biomolecules, NF can be operated in diafiltration (DF) mode [23]. Diafiltration involves the addition of water or any other solvent or buffer to the feed solution to enhance the degree of separation of membrane-retained macrosolutes from membrane-permeable microsolutes [24]. It can be performed in a continuous or discontinuous mode [25]. In continuous diafiltration, the solvent is added to the system at the same rate as permeate flux. Discontinuous diafiltration, instead, involves first diluting the sample with water and then, the diluted sample is concentrated back to its original volume by membrane filtration. Each subsequent dilution should remove more of the small molecules [26]. Typical applications have been studied for the recovery of biochemical products from their fermentation broths [27,28], the purification of water-soluble nanoparticles [29], the reduction of alcohol content from alcoholic beverages [30], the purification of phycocyanin from Spirulina (*Arthrospira maxima*) [31], and the separation of sugars from biologically active compounds [23,32], among many others.

The number of publications dealing with sugar reduction in apple juices (or other natural juices) is very limited. Wei et al. [33] investigated the separation of polyphenols and sugars in apple juice by a loose NF membrane (MWCO 1 kDa) obtaining a sugar-reduced juice as a permeate stream. The results showed that at four bar, the sugar recovery in permeate was around 72% and the recovery of polyphenols in retentate was around 43%. Ceramic tubular UF membranes, with an MWCO of 15 kDa, have recently been investigated to produce a concentrate fraction from apple−cranberry cloudy juice with the simultaneous removal of some amount of sugars [34]. Pruksasri et al. [22] proposed a combination of mechanical pre-fractionation and NF for the reduction of sugar in cloudy apple juice. In the first step, a fruit flesh juice (stream A) with a low content of polyphenols is produced after peeling and coring a certain amount of apple (45% of raw material). In the second step, the flesh juice is treated by NF/diafiltration with the production of a clarified juice (permeate) with low sugar content. The NF permeate is finally mixed with stream B enriched in phenolic compounds produced by milling, pressing, and centrifugation of the rest of the apples together with peels and cores from stream A. Thus, mechanical fractionation was considered as a separation step to reduce the losses of biofunctional compounds.

Bearing in mind that the reduction of sugar in natural fruit juices is a very challenging separation task and its applicability depends on the rejection and selectivity properties of membranes, we investigated an integrated NF/diafiltration approach without peeling apples, thus avoiding any sort of the previous pretreatment. The diafiltration process may contribute to reducing fouling phenomena with a consequent change in selectivity to promote the diffusion of sugars through the membranes. Furthermore, separation characteristics of NF membranes are not only based on size exclusion and this could play in favor of the rejection of polyphenols ahead of sugars during the NF/diafiltration process. In the light of these considerations, the present study evaluated the feasibility of phenolic compounds’ separation from sugars in apple juice by using three different spiral-wound commercial membranes with an MWCO in the range of 200–500 Da. The performance of selected membranes was assessed in terms of productivity (permeate fluxes) and selectivity towards target compounds.

## 2. Materials and Methods

### 2.1. Chemicals

Folin−Ciocalteu phenol reagent, gallic acid, and sodium hydroxide were purchased from Sigma Aldrich (Milan, Italy), while sodium carbonate anhydrous was purchased from Carlo Erba (Milan, Italy). “Yellow line” enzymatic kits were supplied by Roche Diagnostics (Darmstadt, Germany).

### 2.2. Feed Solution

Apple juice was supplied by Gioia Succhi S.r.l. (San Ferdinando, Reggio Calabria, Italy) and processed according to the procedure reported by Gaglianò et al. [21]. The juice was clarified by using a laboratory plant equipped with hollow-fiber UF membranes made of polyethersulfone with an MWCO of 500 kDa (FB-02-FC from Microdyn Nadir, Wiesbaden, Germany). Then, the clarified juice was submitted to a discontinuous diafiltration, followed by an NF process in batch concentration mode. The clarified apple juice composition is reported in Table 1.

### 2.3. Diafiltration−Nanofiltration Process: Experimental Set-Up and Procedure

NF-based diafiltration experiments were performed by using a bench plant (DeltaE S.r.l., Cosenza, Italy) equipped with a stainless steel housing able to accommodate a spiral-wound element with a dimension of 1.8″ × 1.2″ (membrane area of about 0.23 m^2^). The experimental setup consists of a high-pressure pump (Cat Pumps, Milano, Italy, Model 3CP1221), a 5 L stainless steel feed tank, a digital flowmeter (SM6000, ifm electronic gmbh, Essen, Germany), two pressure gauges (Wika Instrument, Lawrenceville, GA, USA), and a control panel. The feed temperature was adjusted by circulating tap water in the two-layered feed tank. The pressure was monitored at the entrance and exit of the membrane module through a frequency inverter and a needle valve located after the membrane module. NF experiments were performed by using three selected spiral-wound membrane modules supplied by Microdyn-Nadir (Wiesbaden, Germany). Their properties are summarized in Table 2.

The diafiltration volume (ratio of the solvent volume added per volume of feed solution) was calculated as follows [38]:

(1) $$DV=\frac{V_{p}}{V_{0}}=\frac{V_{w}}{V_{0}}$$

where *V_p_* and *V*_0_ are the volumes of permeate and feed solution, respectively, and *V_w_* is the volume of water added during the diafiltration process.

The NF process was operated at a transmembrane pressure (TMP) of 25 bar, an axial feed flowrate (Qf) of 7 L/min, and a temperature (T) of 25 ± 1 °C during the diafiltration step. Then, the juice was concentrated according to a batch concentration configuration in the same operating conditions up to a volume reduction factor (VRF) of 4. VRF is defined as the ratio of feed volume to residual retentate volume according to the following equation:

(2) $$VRF=\frac{V_{f}}{V_{r}}=1+\frac{V_{p}}{V_{r}}$$

where *V_f_*, *V_p_*, and *V_r_* are the volume of the feed, permeate, and retentate, respectively.

During both diafiltration and batch concentration steps, the permeate flux was gravimetrically measured at different time intervals.

**Figure 1:**
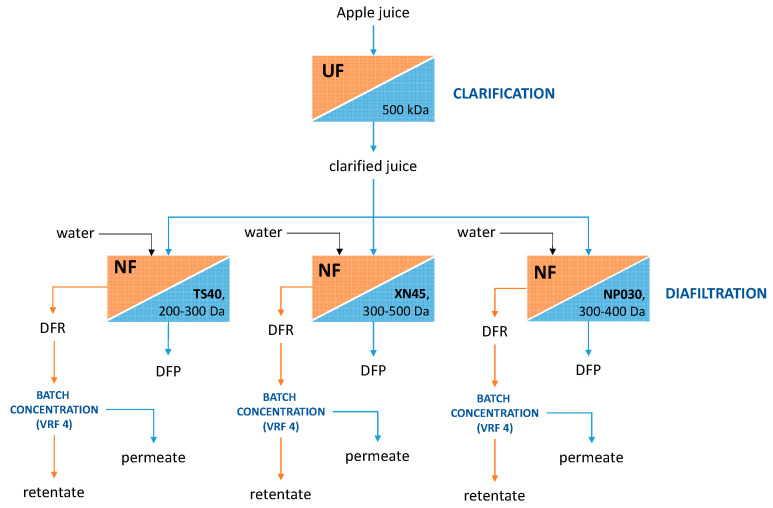
Flow chart of the experimental set-up (UF, ultrafiltration; NF, nanofiltration; DFR, diafiltrated retentate; DFP, diafiltrated permeate).

The flow chart of the experimental set-up is shown in Figure 1.

The fouled membranes were cleaned with an enzymatic solution (Ultrasil 53, 1.0%, 40 °C, 60 min) followed by an alkaline cleaning (Ultraclean WA, 0.2%, 40 °C, 60 min) and final rinsing with tap water. The cleaning efficiency was evaluated by the flux recovery method [39] measuring the water permeability before and after the enzymatic/chemical cleaning.

### 2.4. Performance Parameters

The membrane performance was measured in terms of permeate flux, *J_p_*, concentration factor, *CF_i(R,P)_*, and recovery rate, *R_i(R,P)_* (%), of a certain species.

The volumetric flux of permeate (L/m^2^h) was calculated according to the following equation:

(3) $$J_{p}=\frac{V_{P}}{A\cdot t}$$

where *V_p_* is the volume of collected permeate (L), *t* is the time (h), and *A* the membrane permeation area (m^2^). 

The recovery rate (%) of a species *i* is obtained by its total mass in either permeate or retentate divided by its total mass in feed solution [33,40]:

(4) $$R_{i\left(R,P\right)=\frac{C_{i\left(R,P\right)}\cdot V_{i\left(R,P\right)}}{C_{i\left(F\right)}\cdot V_{i\left(F\right)}}}$$

where *C_i(R,P)_* and *V_i(R,P)_* are the concentration and the volume of species *i* in either permeate or retentate solution, while *C_i(F)_* and *V_i(F)_* are the concentration and the volume of species *i* in the feed. 

The volume *V_iI_* of species *i* in retentate solution and the volume of species *i* in the feed *V_i(F)_* are equal when samples are collected at different DV values during diafiltration. This implies that volumes can be simplified in Equation (4) and the yield values for specific components throughout the diafiltration process can be calculated as a fraction of the original solute concentration remaining in the feed [23]:

(5) $$R_{i\left(R\right)}=\frac{C_{i\left(R\right)}}{C_{i\left(F\right)}}$$

The concentration factor is the concentration of species *i* in either permeate or retentate solution *C_i(R,P)_* divided by its concentration in the feed solution *C_i(F)_* [33]:

(6) $$CF_{i\left(R,P\right)}=\frac{C_{i\left(R,P\right)}}{C_{i\left(F\right)}}$$

The adsorbed phenolics and amount of sugar *Q_ADS_* (mg/m^2^) on the membrane surface at different VRF values was determined as follows [41]:

(7) $$Q_{ADS}=\frac{C_{F}V_{F}-\left(C_{R}V_{R}+C_{P}V_{P}\right)}{A}$$

where *V_F_*, *V_R_*, and *V_P_* are the feed, retentate, and permeate volumes, respectively; *C_R_* and *C_P_* are the concentration of phenolics or sugars in the retentate and permeate streams, respectively, and *A* is the the membrane surface area.

### 2.5. Analytical Measurements

The collected samples include: NF feed, NF retentates at different DV values, NF retentate, and NF permeate at different VRF values. Total soluble solids (TSS), total phenolic content (TPC), D-glucose, and D-fructose were measured. The results of the analytical measurements were expressed as the mean ± standard deviation (SD) of three independent determinations.

#### 2.5.1. Total Soluble Solids 

Total soluble solids (TSS) measurements were carried out by using a hand refractometer (Atago Co., Ltd., Tokyo, Japan) with a scale range of 0–32 °Brix.

#### 2.5.2. Total Phenolic Content

The total phenolic content (TPC) was determined by the Folin−Ciocalteu method [42]. The sample (0.2 mL) was mixed with 1 mL of 10% (*w*/*v*) Folin−Ciocalteu reagent and 0.8 mL of a 7.5% (*w*/*v*) sodium carbonate solution. After 30 min of incubation at room temperature, the absorbance of the resulting solution was measured at 765 nm against a blank. 

Spectrophotometric measurements were performed with the Shimadzu UV-160A UV-visible spectroscopy system. The TPC was calculated on the basis of the calibration curve of gallic acid and expressed as mg of gallic acid equivalents per liter (mg GAE/L).

#### 2.5.3. D-Glucose and D-Fructose Quantification

D-glucose and D-fructose were determined enzymatically in a specific way. A multi-parametric automatic analyzer (iMagic-M9, Darmstadt, Germany) was used to independently perform all the manual analytical procedures required for enzymatic tests. The system automatically collects and dispenses reagents and samples and calculates the analytical data. Detailed procedures used for sample preparation, enzymes, and reactions involved in the determinations of the sugars of interest are reported by R-Biopharm AG [43,44].

## 3. Results and Discussion

### 3.1. Permeate Flux Evaluation

**Figure 2:**
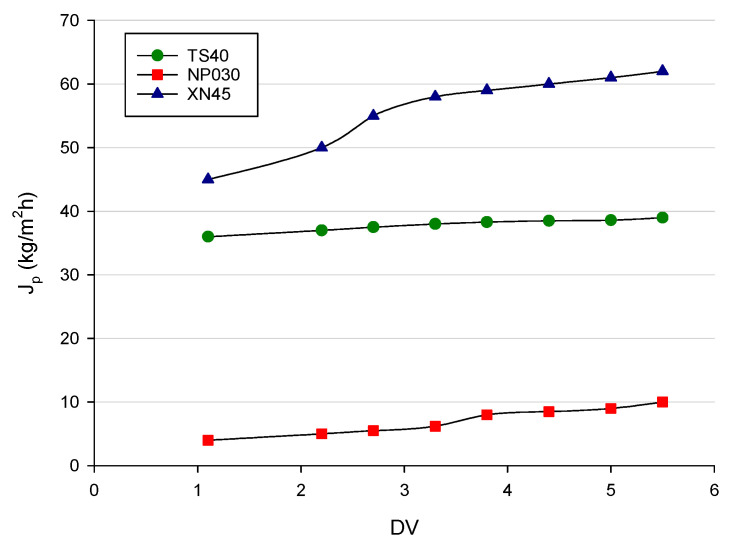
Diafiltration of clarified apple juice with selected membranes. Permeate flux as a function of diafiltration volume (Operating conditions: TMP, 25 bar; Qf, 7 L/min; T, 25 ± 1 °C).

Permeate fluxes and permeate quality are the most important aspects for the selection of a proper membrane [45]. Figure 2 shows the permeate flux (*J_p_*) as a function of diafiltration volumes for all the investigated membranes in the selected operating conditions. For each membrane, the permeate flux increased by increasing the diafiltration volume. The addition of water during the process reduced the viscosity of the feed solution [46] and limited the formation of the concentration polarization layer on the membrane’s surface. Therefore, the clogging of membrane pores was reduced and the transport of microsolutes through the membrane was facilitated [47]. The XN45 membrane exhibited the highest permeate flux with values higher than 60 kg/m^2^h at a DV of 5.5. On the other hand, the NP030 membrane showed much lower fluxes in comparison with TS40 and XN45 membranes, whose greater hydrophilicity played an important role in water solution transport through the membrane [48,49].

**Figure 3:**
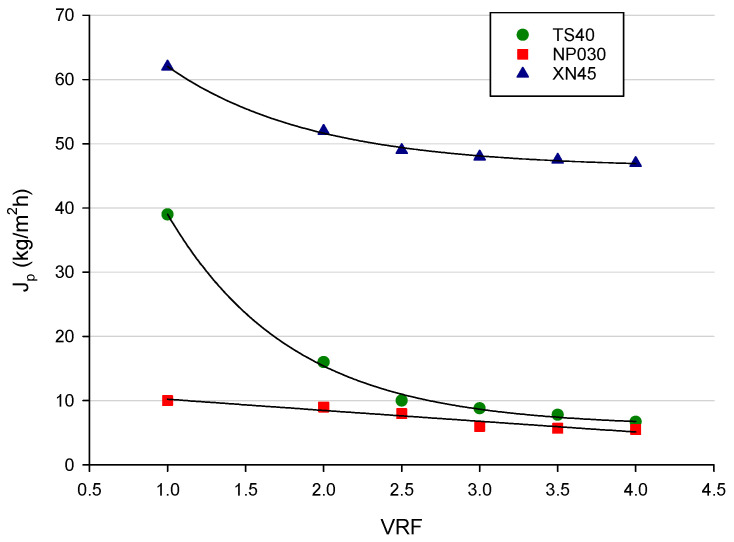
Nanofiltration in batch concentration mode of diafiltered apple juice with selected membranes. Permeate flux as a function of volume reduction factor (Operating conditions: TMP, 25 bar; Q_f_, 7 L/min; T, 25 ± 1 °C).

Figure 3 shows the permeate flux (*J_p_*) as a function of VRF during the NF process of the diafiltered juice with selected membranes according to the batch concentration configuration and in the same operating conditions of the diafiltration process. For XN45 and TS40 membranes, the build-up of solutes at the upstream interface (concentration polarization) determined a rapid permeate flux decay, followed by a long and gradual decline towards a steady-state limit value. Fouling mechanisms, such as the adsorption of particles on the membrane pore walls and pore plugging, are additional phenomena [50]. In the selected operating conditions, steady-state values for XN45 and TS40 membranes were in the order of 48 kg/m^2^h and 8 kg/m^2^h, respectively. For the NP030 membrane, the initial permeate flux was about 10 kg/m^2^h, much lower than those observed for the other two investigated membranes. In addition, for this membrane, the permeate flux reduction at VRF 4 was only about 20% in relation to the initial value.

For all selected membranes, the cleaning efficiency after the cleaning protocol was higher than 90%.

### 3.2. Recovery Rate of D-Glucose, D-Fructose, and TPC during Discontinuous Diafiltration

**Figure 4:**
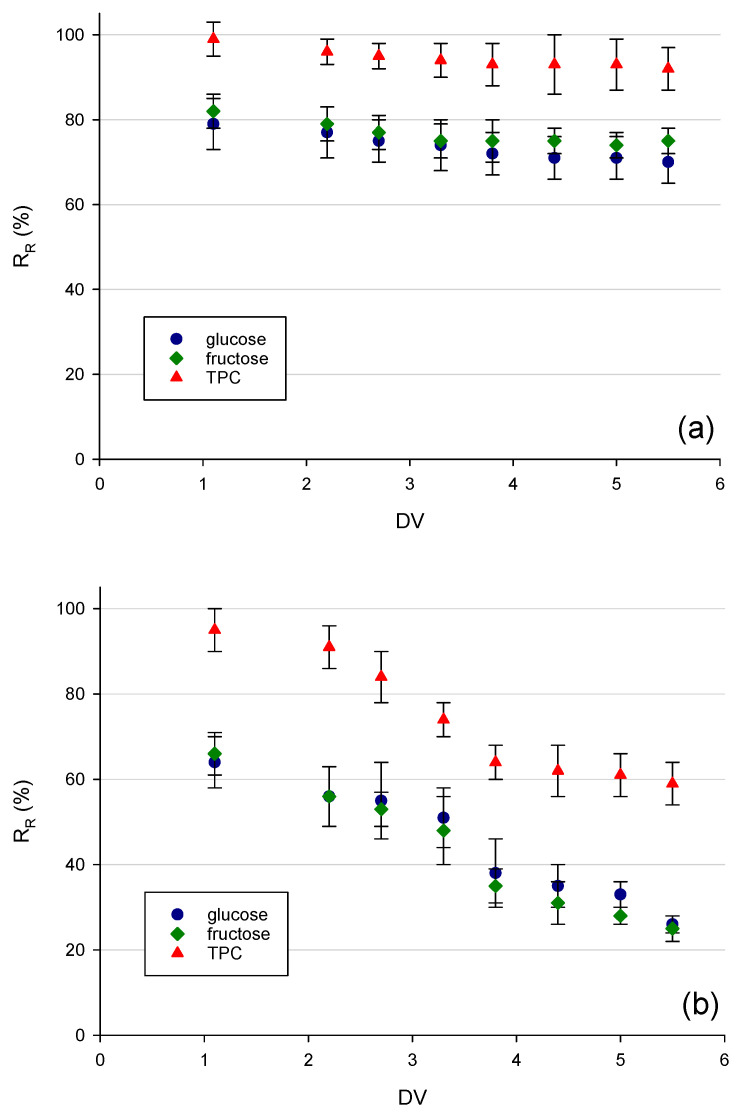

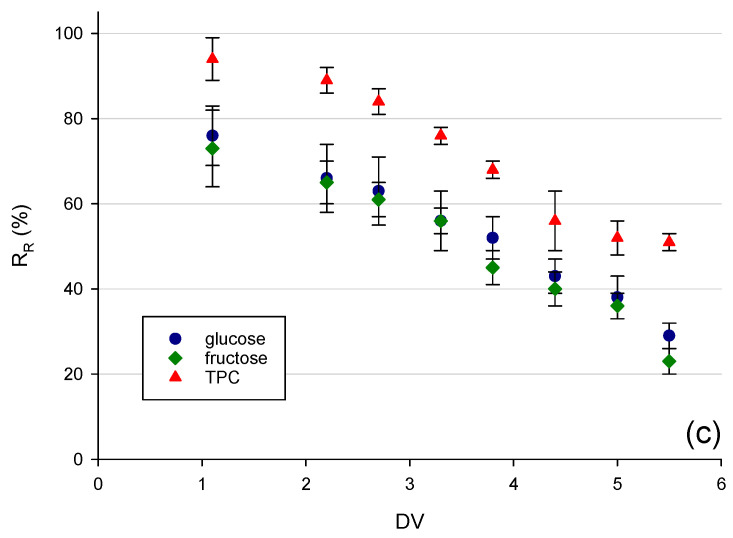
Recovery rate of total phenolic compounds, glucose, and fructose in the retentate stream during diafiltration with (**a**) TS40 (**b**) NP030 and (**c**) XN45 membranes.

The recovery rate of both phenolic compounds and carbohydrates in the retentate stream was plotted as a function of the diafiltration volume, as shown in Figure 4. The selected membranes showed a similar behaviour: in particular, the recovery rate of both sugars and TPC decreased in the retentate by increasing the diafiltration volume indicating that the addition of distilled water as washing solvent during the diafiltration process caused the removal of these compounds in the clarified extract. Nevertheless, carbohydrates and TPC were removed at varying degrees for different membranes. Regardless of the diafiltration volume, the separation factor between TPC and sugars remained almost unchanged during the process: in fact, the more the number of diavolumes increased, the more the sugars passed into the permeate in the same ratio at which the polyphenols also passed. This has both negative and positive aspects. The negative aspect is linked to the fact that the separation between the compounds in the selected operating conditions cannot be improved with the addition of water, but this also represents an advantage from an economic point of view because the separation reached at DV 5.5 is almost the same as that obtained at DV 1.1. This implies a lower consumption of fresh water is needed to achieve the same result.

Although the NP030 membrane was characterized by lower permeate fluxes, it allowed for the achievement of a greater separation factor between TPC and carbohydrates. At DV of 1.1, nearly 36% of D-glucose and 34% of D-fructose were removed, while 95% of TPC remained in the retentate juice. According to Pruksasri et al. [22], a degree of sugar reduction of about 30–40% could be an appropriate target. Therefore, a single diananofiltration process accomplished with an appropriate membrane and proper selected operating conditions may be applicable to remove a sufficient amount of sugar and efficiently recover phenolic compounds without losing their activity. In this way, a diafiltered retentate as the high-added value product can be obtained with significant advantages from both nutritional and economical points of view.

### 3.3. Concentration Factor of D-Glucose, D-Fructose, and TPC during Nanofiltration in Batch Concentration Mode

**Figure 5:**
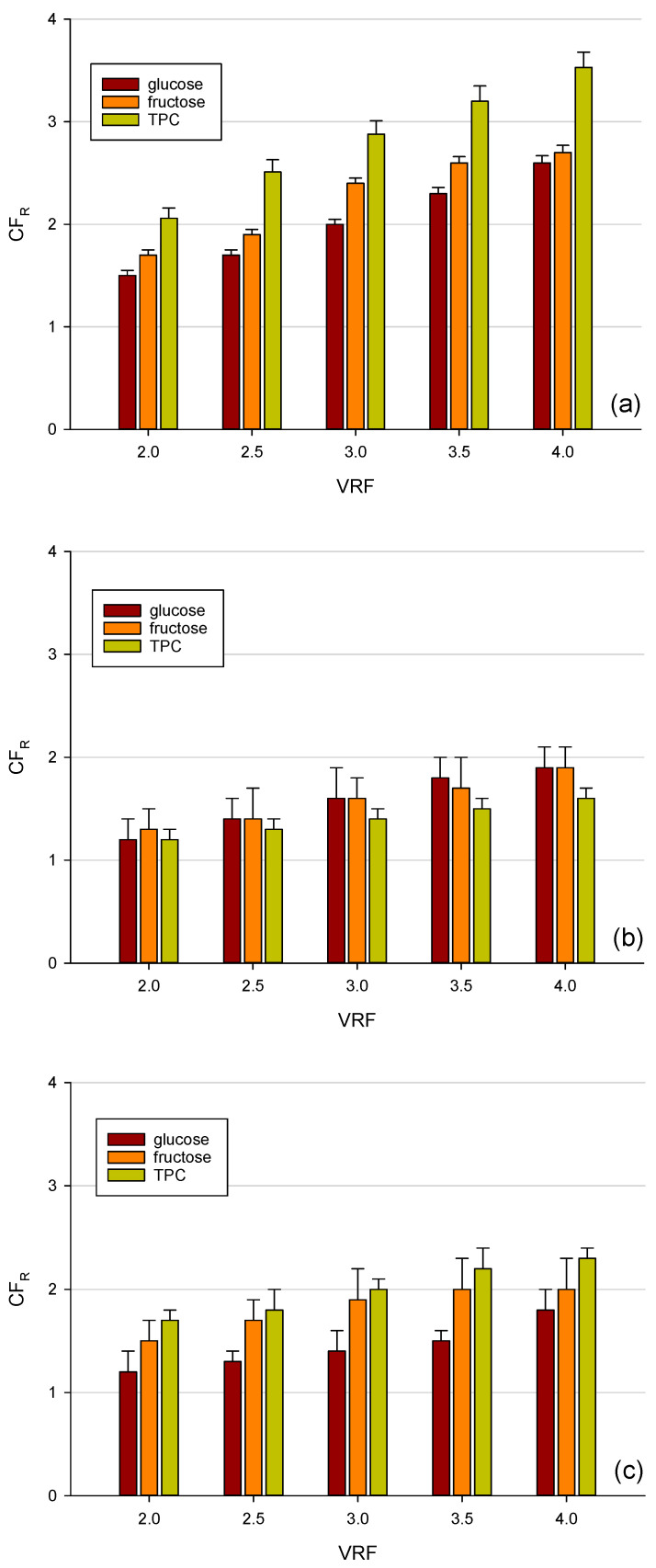
Concentration factor of total phenolic compounds, glucose, and fructose in the retentate stream during the concentration with (**a**) TS40 (**b**) NP030 and (**c**) XN45 membranes.

After diafiltration, the clarified juice was concentrated up to VRF 4 with each one of the investigated membranes. In this way, it was possible to evaluate the behaviour of each membrane in retaining and separating the compounds of interest in concentration mode. It is known that rejection is controlled by molecular size and interaction force between the membrane and the solute [51]. Among the investigated membranes, the TS40 had a higher rejection of sugars and polyphenols due to its lower MWCO of 200–300: in fact, the concentration factor at VRF 4 was 3.5 for TPC and 2.6 for sugars, respectively (Figure 5a). Therefore, only about 12.5% of the polyphenols were lost during the batch concentration process, while about 35% of sugars were further removed. The XN45 membrane showed the same trend: a higher concentration factor for polyphenols was observed in comparison to glucose and fructose. However, this membrane exhibited the worst separation gap between carbohydrates and TPC and a major removal in the retentate sample for all compounds. The concentration factor at VRF 4 was 2.3 for TPC, 2.0 for fructose, and 1.6 for glucose, respectively (Figure 5c). On the contrary, for the NP030 membrane, the concentration factor was higher for glucose and fructose than polyphenols, independently of the VRF value.

For all selected membranes, the retention level was relatively high when compared with the molecular weight of glucose (180.1559 g/mol) and fructose (180.16 g/mol). These results can be explained assuming that other phenomena, in addition to the steric hindrance, affect the retention coefficients including interactions between the solute and membrane material, as well as the association of sugars with phenolic compounds [52].

In addition, for all selected membranes, a significant increase in phenolic compounds was observed by increasing the VRF of the process. An increased concentration of phenolic compounds as a function of the VRF of the process was also observed in the treatment of mate bark aqueous extracts [53] and artichoke brine [54], with spiral-wound NF membranes characterized by an approximate MWCO of 150–300 Da (Desal HL and Desal DG from GE Osmonics, respectively). Different factors, including the surface morphology, pore size distribution, and adhesion in the membrane may contribute to this phenomenon [55].

**Figure 6:**
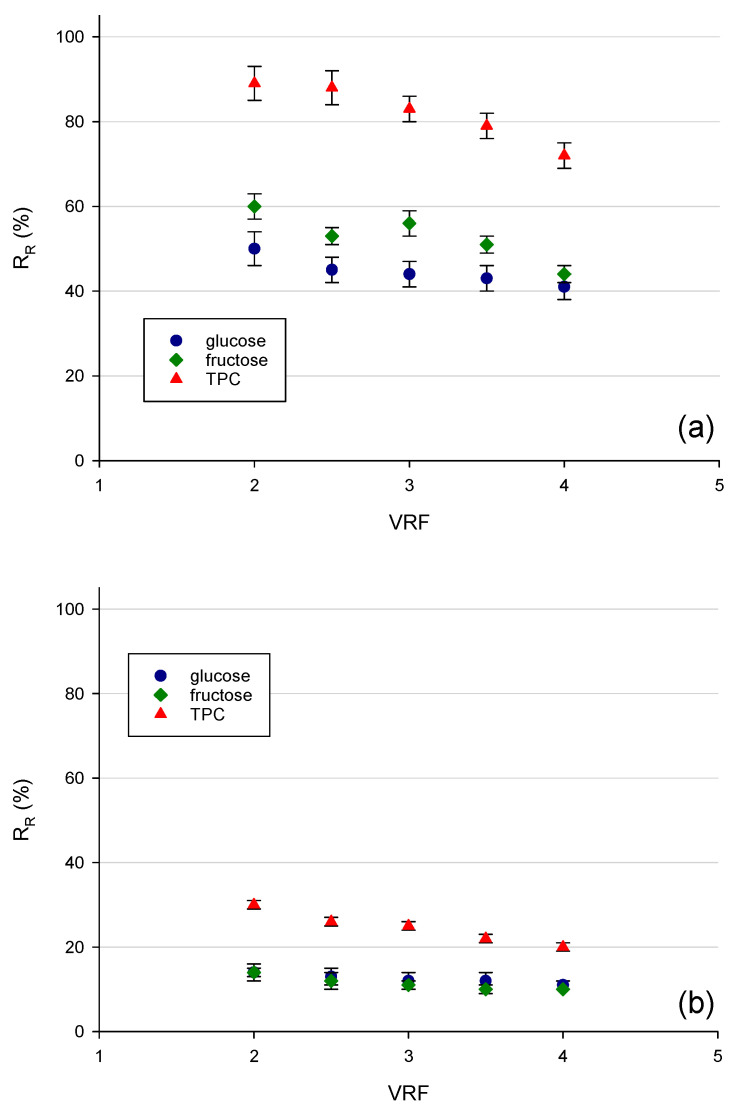

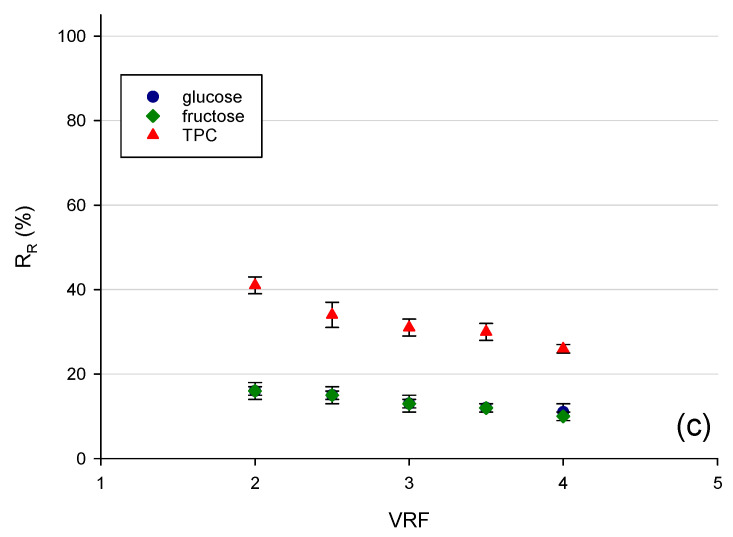
Recovery rate of total phenolic compounds, glucose, and fructose in the retentate stream during the batch concentration process with (**a**) TS40 (**b**) NP030 and (**c**) XN45 membranes.

In Figure 6, the recovery rate of TPC, glucose, and fructose in the retentate stream as a function of VRF for all selected membranes is shown. At VRF 4, more than 70% of phenolic compounds were recovered in the retentate of the TS40 membrane; at the same time, the removal efficiency of glucose and fructose was 59% and 56%, respectively (corresponding to a recovery factor in the retentate of 41% and 44%, respectively).

For membranes NP030 and XN45, the recovery factors of both phenolic compounds and sugars in the retentate stream were significantly lower than those observed for the TS40 membrane. Indeed, for the NP030 membranes, the recovery factors of sugars and phenolic compounds were about 10% and 20%, respectively. Similar values were observed for the recovery of sugar compounds in the retentate of the XN45 membrane while for phenolic compounds, the recovery factor at VRF 4 was 26%. Therefore, the global result indicated the TS40 membrane was the best one to preserve phenolic compounds of apple juice through a combination of diafiltration and batch concentration processes while reducing the content of sugars by an order of 60%.

### 3.4. Mass Balance and Adsorption of Sugars and TPC

The mass balance of the whole filtration process, which includes the initial feed solution, the diafiltrated solution, the permeate stream, and the remaining retentate can give an insight into deposit formation on the membrane surface and eventual pore blocking. Table 3 shows the mass balance related to sugars and TPC in both diafiltration and concentration processes for the selected membranes. As it can be seen, the batch concentration configuration is characterized by a substantial deviation from the mass balance in comparison to diafiltration. These results can be attributed to pore blocking phenomena due to the adsorption of monosaccharides and low molecular weight phenolic compounds on both the outer and inner membrane surface.

The TS40 membrane showed the lowest deviation from the mass balance of TPC during nanofiltration in concentration mode. This might be due to the smallest MWCO of this membrane which tends to prevent the inner pore blocking of polyphenols. On the other hand, the mass balance of both sugars and TPC in the diafiltration process was higher than 91% for all selected membranes. Diafiltration ensures more favorable hydrodynamic conditions in the membrane boundary layer, thus controlling the thickness of the concentration polarization layer and pore-clogging. These conditions reduce the amount of sugar adsorbed on the membrane, improving their diffusion. Similar results have been reported recently by Tonova et al. [56] who evaluated the performance of the NP030 membrane in flat-sheet configuration in the separation of saccharides from phenolics from an aquatic weed hydrolysate.

**Figure 7:**
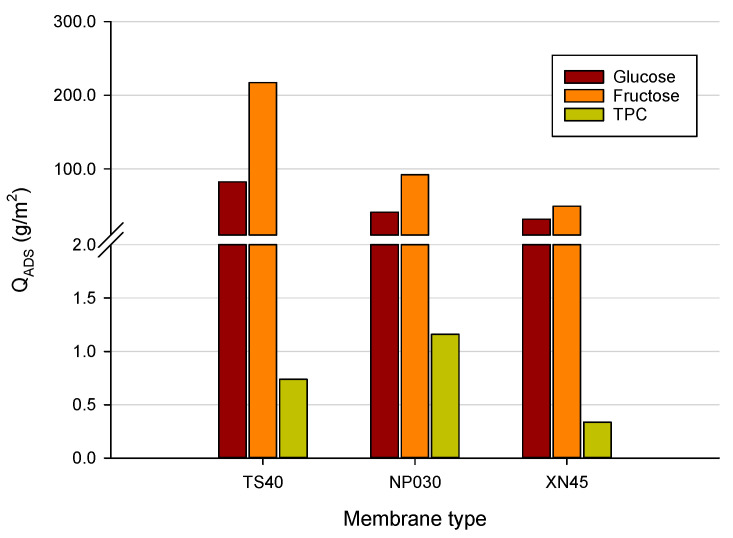
Amount of adsorbed glucose, fructose, and TPC for selected membranes.

In Figure 7 the adsorbed amount of sugar and TPC on the selected membranes evaluated at the end of the batch concentration process is reported. The amount of adsorbed fructose for the TS40 membrane, about 217 g/m^2^, was much higher in comparison to the other NF membranes. The adsorbed amount of glucose for this membrane (82.39 g/m^2^) was also higher than the other two membranes (30.13 and 31.73 g/m^2^ for NP030 and XN45 membranes, respectively). On the other hand, the adsorbed amount of phenolic compound for all selected membranes was much lower than that of sugars in relation to the predominant content of sugars in the original juice. Among the selected membranes, the NP030 exhibited the highest amount of adsorbed phenolics (1.16 g/m^2^ against 0.74 and 0.33 g/m^2^ for TS40 and XN45 membranes, respectively). Saleh et al. [57] reported up to 4.3 g of polyphenolics adsorbed per m^2^ of the membrane in the treatment of apple juice with a SelRO^®^ spiral-wound membrane of 1 kDa MWCO (MPS-36 from Koch Membrane Systems).

The more hydrophobic character of the NP030 membrane, with a top layer in PES material, enhances the adsorption of phenolic compounds on the membrane surface and the formation of a hydrophobic layer onto the membrane surface which could modify the physicochemical properties of the membrane [58], leading to lower permeate fluxes and modification of the rejection performance. Susanto et al. [59] supported the existence of benzene ring−benzene ring interactions to justify the adsorption of phenolic compounds on PES membranes. Moreover, the formation of multiple hydrogen bonds between polyphenols and the additive poly(vinyl)pyrrolidone (PVP) usually used in the manufacturing process in PES membranes is considered an additional contribution to the adsorption phenomena of phenolic compounds [60].

Cai et al. [61] found that for phenolic compounds, a cake/gel layer as a reversible fouling was the main fouling resistance, while the adsorption played a significant role in the irreversible fouling resistance of polyamide and polypiperazine amide NF membranes with MWCOs of 240 and 150 Da, respectively.

## 4. Conclusions

Nanofiltration membranes with an MWCO in the range of 200–600 Da were tested to reduce the sugar content in apple juice through a combination of diafiltration and batch concentration processes. For all selected membranes, the recovery rate of both sugars and phenolic compounds decreased in the retentate by increasing the diafiltration volume; however, the separation factor between phenolic compounds and sugars remained almost unchanged during the diafiltration process.

Among the investigated membranes, the TS40 membrane, a thin-film composite membrane with the lowest MWCO (200–300 Da), showed the highest retention of sugars and phenolic compounds in the selected operating conditions of both diafiltration and concentration processes. More than 70% of phenolic compounds were recovered in the retentate stream of this membrane at a volume reduction factor of 4, while recoveries of glucose and fructose were 41 and 44%, respectively. On the other hand, the other investigated membranes allowed for the recovery of a much lower amount of phenolic compound in the retentate stream despite a greater removal of sugars on the permeate side. Therefore, the global results indicate that the combination of diafiltration and batch concentration with the TS40 membrane is a good compromise to remove up to 60% of sugars from apple juice with minimal losses of phenolic compounds.

## Figures and Tables

**Table 1:** Composition of clarified apple juice subjected to a diafiltration−nanofiltration process.

Parameter	Value
Glucose (g/L)	17.2 ± 1.3
Fructose (g/L)	43.7 ± 2.4
Total phenolic content (mgGAE/L)	241.5 ± 8.1
Total soluble solids (°Brix)	7.0 ± 0.1
pH	3.78 ± 0.02

**Table 2:** Characteristics of selected membranes according to manufacturers unless otherwise stated.

Membrane Type	TS40	XN45	NP030
Membrane material	TFC	TFC	PES
Configuration	spiral-wound	spiral-wound	spiral-wound
Max. operating pressure (bar)	41	41	35
Max. operating temperature (°C)	50	50	70
pH	1–12	1–12	0–14
Membrane surface area (m^2^)	0.23	0.23	0.23
Nominal MWCO (Da)	200–300	300–500	300–400
Contact angle (°)	30 ^a^	57 ^b^	80 ^c^
Water permeability at 25 °C (kg/m^2^hbar)	4.48 ^d^	6.12 ^d^	2.99 ^d^

TFC, thin-film composite, PES, polyethersulphone; ^a^ data from Zdarta et al. [35]; ^b^ data from Peiris et al. [36]; ^c^ data from Boussu et al. [37]; ^d^ own measurements.

**Table 3:** Mass balance (%) of glucose, fructose, and TPC in diafiltration and batch concentration processes for selected membrane.

Membrane Type	Process	Component		
		Glucose	Fructose	TPC
TS40	Diafiltration	94.62	93.56	97.49
	Batch concentration	68.81	68.19	81.51
NP030	Diafiltration	95.83	99.99	90.98
	Batch concentration	59.07	59.63	54.33
XN45	Diafiltration	100.00	99.99	99.99
	Batch concentration	63.13	72.45	58.00

## Data Availability

The data presented in this study are available upon request from the corresponding author.
